# Safety and efficacy of ERCP using standard duodenoscopes in pediatric patients with choledocholithiasis: A retrospective study

**DOI:** 10.1097/MD.0000000000048707

**Published:** 2026-05-08

**Authors:** Berk Baş, Ömer Küçükdemirci, Müge Ustaoğlu, Ahmet Bektaş

**Affiliations:** a Department of Gastroenterology and Hepatology, Adnan Menderes University, School of Medicine, Aydin, Türkiye; b Department of Gastroenterology and Hepatology, Hakkari State Hospital, Hakkari, Türkiye; c Department of Gastroenterology and Hepatology, Ondokuz Mayis University, School of Medicine, Atakum/Samsun, Türkiye.

**Keywords:** biliary tract, cannulation, choledocholithiasis, complications, duodenoscope, ERCP, pediatric ERCP

## Abstract

This study aimed to evaluate the safety and efficacy of endoscopic retrograde cholangiopancreatography (ERCP) in children aged 2 to 13 years with choledocholithiasis, addressing the challenges of using standard adult duodenoscopes in a pediatric population. Materials and Methods: We retrospectively reviewed the medical records of 107 patients who underwent ERCP at a single tertiary ERCP center between 2014 and 2024. Among these, patients with chronic pancreatitis or biliary strictures were excluded, resulting in a final cohort of 70 pediatric patients. A control group of 397 adult patients with similar conditions was analyzed for comparison. Procedural success was defined as successful deep cannulation of the common bile duct and stone extraction. The pediatric group achieved a procedural success rate of 91.4%, compared to 95.2% in adults, with no statistically significant difference (*P* = .278). Complication rates were low and similar between groups: bleeding occurred in 8.6% of pediatric patients, while pancreatitis was noted in 1.4%. No mortalities were reported in the pediatric cohort. The findings indicate that ERCP can be performed safely and effectively in children using standard adult duodenoscopes by experienced adult gastroenterologists under proper supervision. The procedural success rates and complication rates were comparable to adults, suggesting that ERCP is a viable option for pediatric choledocholithiasis. To optimize outcomes, further multicenter studies and improved pediatric training are recommended.

## 1. Introduction

Endoscopic retrograde pancreatography (ERCP) has been widely used in the diagnosis and treatment of various malignant and benign biliary tract diseases in the adult population since the 1960s, with an acceptable rate of complications.^[1]^ However, in the pediatric population, the use of this method has been less frequent due to the relatively higher technical difficulty of the procedure and the limited experience of pediatric gastroenterologists in this field. With the development of pediatric-specific duodenoscopes in the late 1980s, these challenges were partially overcome, and ERCP became applicable in children as well.^[2,3]^ Nevertheless, due to the lack of pediatric duodenoscopes in most centers and the limited experience of pediatric gastroenterologists, children requiring this procedure are generally referred to a small number of advanced centers.^[4,5]^

In cases where referral to advanced centers is not possible, pediatric gastroenterologists manage pediatric patients in emergency situations using standard adult duodenoscopes.^[6,7]^ However, the success rate of such procedures and the associated complication rates have been evaluated in the literature through only a limited number of studies with restricted scope.

Currently, there are no widely accepted definitive indications for endoscopic retrograde cholangiopancreatography (ERCP) in pediatric populations.^[7]^ Nonetheless, children are frequently subjected to ERCP for indications that closely mirror those established in adults. One unequivocal and undisputed indication is the presence of gallstones causing cholangitis, jaundice, or chronic pain.^[7–9]^ Although the literature includes numerous studies on pediatric ERCP, specific investigations focusing on gallstones – the most common and precise indication – and procedures performed using standard duodenoscopes by adult gastroenterologists remain scarce. Such studies are often limited to subgroups within broader analyses.^[9,10]^ In the present study, we concentrated on this particular patient cohort to address this gap.

## 2. Material and method

**Figure 1.**
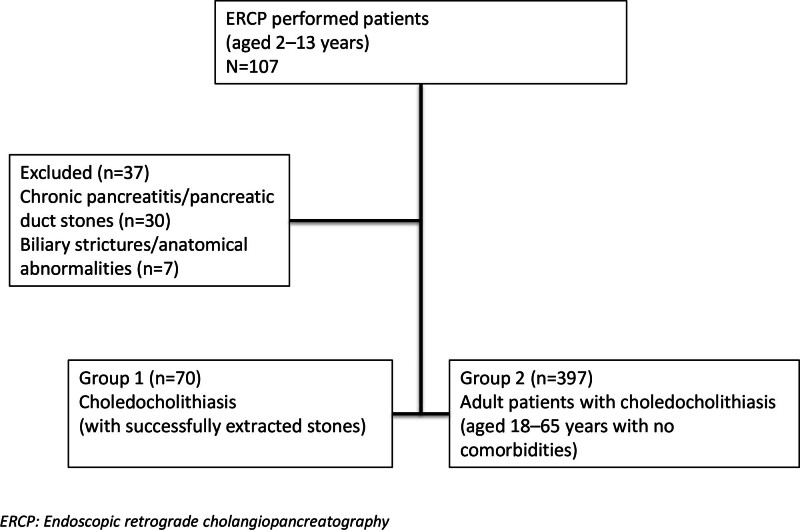
Flow chart of participants.

This study retrospectively reviewed the medical records of 107 patients aged 2 to 13 years who underwent ERCP at the Adult Gastroenterology Clinic of Ondokuz Mayis University Hospital between 2014 and 2024. Among these, 30 patients who underwent the procedure due to chronic pancreatitis or pancreatic duct stones, and 7 patients in whom biliary strictures or anatomical abnormalities were detected without the presence of stones, were excluded from the study (Fig. 1). These patients were excluded to create a more homogeneous study population focused on choledocholithiasis and to avoid potential confounding factors, as chronic pancreatitis, pancreatic duct stones, and anatomical abnormalities may require different ERCP techniques and therapeutic approaches and may be associated with different complication risks.

A total of 70 (51 females, 19 males) patients in whom choledocholithiasis was identified and the stone was successfully extracted during the procedure were included in the study. Patients aged 13 to 18 years were excluded, as they may exhibit adult physiological characteristics. As the control group (Group 2), 397 adult patients (230 females, 167 males) aged 18 to 65 years who were diagnosed with choledocholithiasis (Table 1), underwent ERCP during the same period at the same clinic, and had no additional comorbidities, were included for comparison with the pediatric ERCP group (Group 1). Informed consent regarding the procedure was obtained from all patients’ legal guardians or from the patients themselves. Ethical approval for the study was obtained from the Clinical Research Ethics Committee of Ondokuz Mayis University.

**Table 1:** Gender distribution by age groups.

	Female	Male	Total
Children	51	19	70
Adult	167	230	397

### 2.1. Procedure

All patients had choledocholithiasis confirmed by at least 1 imaging modality, such as transabdominal ultrasonography (US), endoscopic ultrasonography (EUS), computed tomography (CT), or magnetic resonance cholangiopancreatography (MRCP). All procedures were performed under deep sedation on a fluoroscopy table, with the assistance of an anesthesiologist, by 5 adult gastroenterologists, each with at least 10 years of experience in the field, using Olympus TJF-260V Duodenoscope (Olympus Co., Japan) with a outer diameter of 11.6 mm and a working length of 1240 mm. In our institution, ERCP is performed in a high-volume tertiary referral center. Endoscopists are not permitted to perform ERCP independently unless they have substantial procedural experience, typically requiring experience with at least 200 ERCP procedures under supervision. In pediatric patients, all procedures were performed by experienced adult gastroenterologists under the supervision of a pediatrician and/or pediatric gastroenterologist to ensure appropriate pediatric assessment and monitoring. In all procedures, procedural success was defined as the deep cannulation of the common bile duct using a conical-tipped guidewire following the cannulation of the ampulla of Vater with either a standard pull-type sphincterotome or a needle-type sphincterotome.

### 2.2. Statistics

Statistical analyses were conducted using IBM SPSS Statistics Version 29. Descriptive statistics were presented as counts and percentages for categorical variables. Associations between categorical variables and group classifications were examined using cross-tabulations with Pearson’s chi-square tests, including continuity correction and Fisher’s exact tests when appropriate. Significance levels were set at *P* < .05. Multiple correspondence analysis was performed to explore relationships between categorical variables and their underlying dimensions, with normalization by variable principal normalization and a maximum of 100 iterations to ensure convergence. The variance accounted for by dimensions was reported along with Cronbach’s alpha to assess internal consistency. All analyses accounted for missing data by treating user-defined missing values as missing, and only cases with complete data for the variables of interest were included. Graphical representations such as bar charts were used to illustrate distributions and associations.

## 3. Results

The study cohort included 70 pediatric patients (age 2–13 years) and 397 adult patients (age 18–65 years). In the pediatric group, 51 patients (72.9%) were female and 19 (27.1%) were male. In the adult group, 167 patients (42.1%) were male and 230 patients (57.9%) were female (Table 1).

Procedural success (defined as deep common bile duct cannulation and stone extraction) was achieved in 64 of 70 pediatric patients (91.4%) and in 378 of 397 adult patients (95.2%); this difference was not statistically significant (*P* = .278). Procedure repetition was required in 5 of 6 indicated pediatric cases and in 12 of 21 indicated adult cases (*P* = .908). Fistulotomy was performed in 8/70 pediatric patients (11.4%) and 44/397 adult patients (11.1%) (*P* = .284). Precut sphincterotomy was applied in 8/70 children (11.4%) and 77/397 adults (19.4%) (*P* = .908). Pancreatic duct cannulation occurred in 3/70 children (4.3%) and 39/397 adults (9.8%) (*P* = .135). In pediatric patients, pancreatic duct cannulation occurred unintentionally during attempts at biliary cannulation. In 1 patient, a prophylactic pancreatic duct stent was placed due to repeated pancreatic duct cannulation in order to reduce the risk of post-ERCP pancreatitis. Similarly, pancreatic stents were also placed in adult patients for the same indication when repeated pancreatic duct cannulation was observed. Percutaneous transhepatic biliary cannulation (PTC) was not required in pediatric patients (0/70) and was required in 8/397 adults (2.0%) (*P* = .287). Overall, no statistically significant differences were observed between groups for these procedural measures (Table 2).

**Table 2:** Complications between children and adult patients.

Complication	Children	Adults	*P* value
Hemorrhage	6/70	18/397	0,667
Pancreatitis	1/70	18/397	0,225
Colangitis	1/70	16/397	0,284
Perforation	1/70	5/397	0,908
Surgical intervention	0/70	5/397	0,908
Intensive care requirement	1/70	16/397	0,087
Mortality	0/70	1/397	0,674

Overall complication rates did not differ significantly between pediatric and adult patients. In the pediatric group (n = 70), bleeding occurred in 6 patients (8.6%), pancreatitis in 1 patient (1.4%), cholangitis in 1 patient (1.4%), perforation in 1 patient (1.4%), and 1 patient (1.4%) required intensive care. There were no surgical interventions or mortalities in the pediatric group. In the adult group (n = 397), bleeding occurred in 18 patients (4.5%), pancreatitis in 18 patients (4.5%), cholangitis in 16 patients (4.0%), perforation in 5 patients (1.3%), 5 patients (1.3%) required surgical intervention, 16 patients (4.0%) required intensive care, and there was 1 death (0.25%). None of the between‑group differences reached statistical significance (bleeding *P* = .667; pancreatitis *P* = .225; cholangitis *P* = .284; perforation *P* = .908; need for surgery *P* = .908; intensive care *P* = .087; mortality *P* = .674) (Table 3).

**Table 3:** Procedural outcomes between children and adult patients.

Procedural outcome	Children	Adults	*P* value
Procedure success	64/70	378/397	0,278
Procedure repetition	5/6	12/21	0,908
Precut	8/70	77/397	0,908
Pancreatic cannulation	3/70	39/397	0,135
Percutaneous transhepatic biliary cannulation	0/70	8/397	0,287

Common bile duct stents were placed in 8 of 70 pediatric patients (11.4%) and in 65 of 397 adult patients (16.4%) (*P* = .294). Pancreatic stents were placed in 1/70 children (1.4%) and in 14/397 adults (3.5%) (*P* = .225). These differences were not statistically significant (Table 4).

**Table 4:** Comparison of stenting outcomes in children vs adults.

	Children	Adult	*P* value
Biliary stent	8/70	65/397	0,294
Pancreatic stent	1/70	14/397	0,225

## 4. Discussion

In the pediatric population, the use of Endoscopic Retrograde Cholangiopancreatography (ERCP) has been limited due to a combination of factors, including a low incidence of conditions necessitating the procedure and the perception that it is technically challenging in children.^[11,12]^ While there has been an increase in the need for ERCP over the past 2 decades, pediatric gastroenterologists often lack sufficient training in advanced endoscopic techniques, leading to reliance on adult gastroenterologists who may not have access to pediatric-specific equipment.^[13,14]^ The existing literature reveals a significant gap in data concerning ERCP outcomes in children compared to adults, with most guidelines and complication rates being well-established for the adult population. This disparity is further complicated by the rarity of pancreatic and biliary diseases in children, which limits the frequency of these procedures among pediatric specialists. Despite these challenges, recent studies indicate that ERCP can be performed safely in pediatric patients using standard duodenoscopes, suggesting that with proper training and resources, the efficacy of this procedure can be improved for younger patients. In many centers worldwide, ERCP in pediatric patients is performed by experienced adult gastroenterologists, particularly in high-volume ERCP units where pediatric-specific equipment or specialized pediatric ERCP expertise may not always be available.^[14–16]^ In our center, pediatric ERCP procedures were performed by adult gastroenterologists with extensive ERCP experience and under the supervision of a pediatrician and/or pediatric gastroenterologist, ensuring both technical expertise and appropriate pediatric monitoring. Furthermore, institutional credentialing policies in our center require substantial ERCP experience before independent practice, which further supports procedural safety in both adult and pediatric patient populations.

The literature contains varying findings regarding the complication rates associated with endoscopic retrograde cholangiopancreatography (ERCP) procedures in pediatric patients. Large-scale meta-analyses, primarily conducted by adult gastroenterologists, report data indicating no significant differences in complication rates between children and adults.^[17]^ In contrast, a recently published study evaluating 17 years of clinical data from pediatric gastroenterology centers suggests that complication rates are higher in children. These conflicting results contribute to the ongoing discourse on the safety and efficacy of pediatric ERCP procedures.^[18]^ In our study, all procedures were performed by adult gastroenterologists skilled in endoscopic retrograde cholangiopancreatography (ERCP), under the supervision of a pediatrician and/or pediatric gastroenterologist, and with deep sedation administered by an anesthesiologist. No pediatric patient undergoing ERCP for biliary stone complications experienced mortality or required subsequent surgical intervention. Intensive care unit admission was necessary in only 1 case. There was 1 case each of pancreatitis, cholangitis, and perforation. Six patients developed bleeding, which was successfully controlled. When compared to the adult group, no significant differences were found regarding bleeding, perforation, the need for surgery, or mortality. To minimize the occurrence of additional complications and procedural failures, the control group was selected from adults aged 18 to 65 years without comorbidities who underwent ERCP for biliary stone indications, nevertheless, all other complications were observed less frequently in the pediatric age group.

Many studies evaluating ERCP success in pediatric patients include an age range of 1 to 18 years; however, a commonly cited limitation and point of criticism in these studies is that children aged 13 to 18 often exhibit adult physiological characteristics.^[10,16,19]^ To address this issue and minimize the influence of adult-like features on procedural outcomes, we established an age cutoff of 13 years in our study to better represent a pediatric population with fewer adult characteristics during outcome assessment. Successful cannulation of the desired biliary duct is 1 of the most critical and challenging steps for achieving a successful ERCP.^[20]^ Mastery of cannulation techniques is a fundamental component of ERCP training. For an adult gastroenterologist to perform ERCP independently, it is generally accepted that completing at least 160 to 200 procedures is necessary to attain technical competency.^[21]^ A meta-analysis has indicated that real-world cannulation rates typically range from the high 80s to 90%, although documented variations exist globally.^[22]^ In our study, the success rates of biliary cannulation, considered as procedural success, are consistent with those reported in the literature. Moreover, we separately evaluated cases in which the initial procedure was unsuccessful but success was achieved during a subsequent attempt. According to this approach, 69 out of 70 pediatric cases achieved successful biliary cannulation by the first or second procedure, slightly outperforming our own adult control group, and also exceeding the success rates commonly reported in the literature.

In adults, cannulation of the pancreatic duct is generally easier than biliary duct cannulation due to the anatomical angle at which the pancreatic duct enters the hepatopancreatic ampulla.^[23]^ Consequently, pancreatic cannulation frequently occurs in many cases. However, this rate decreases with increased endoscopist experience; notably, centers performing a high-volume of ERCP procedures report lower rates of pancreatic cannulation and pancreatic stenting.^[24]^ In contrast, there is a lack of data regarding false pancreatic cannulation in the pediatric population. In our study, both false cannulation and pancreatic stenting rates were found to be lower in children compared to our adult control group. The rate of biliary stent placement in adults was approximately twice as high as in children. This difference may be attributed to the fact that adults more frequently have larger stones lodged in the duct that cannot be removed easily, necessitating stent placement to ensure ductal patency.^[25]^ In our study, pancreatic duct cannulation observed in a small number of pediatric patients occurred unintentionally during attempts at biliary cannulation, which is a known phenomenon during ERCP due to papillary anatomy. In 1 pediatric patient, a prophylactic pancreatic duct stent was placed following repeated pancreatic duct cannulation to reduce the risk of post-ERCP pancreatitis.

This study has several important limitations. Firstly, due to its retrospective design, there may have been some incompleteness and uncertainties in data collection. The study was conducted at a single center, which may limit the generalizability of the results, as the patient population represents a specific geographic and socio-economic group. Additionally, adult endoscopists performing ERCP procedures in both pediatric and adult patients have over 10 years of experience and operate in a high-volume ERCP center, which may make them more experienced than the average endoscopist population; this factor could have influenced the outcomes. The technical limitations and equipment incompatibilities associated with the use of standard adult duodenoscopes in the pediatric age group also represent factors that may affect procedural success. Furthermore, the limited data in the literature regarding pancreatic duct cannulation and complications in children make comparative evaluation of our findings more challenging. Finally, the exclusion of adolescents over 13 years of age from the study resulted in a lack of information concerning the transitional period between pediatric and adult patient groups. Considering these limitations, our findings should be supported by larger, multicenter studies to enhance their validity and applicability.

## 5. Conclusion

This study demonstrates that endoscopic retrograde cholangiopancreatography (ERCP) can be performed safely and effectively in the pediatric population aged 2 to 13 years using standard adult duodenoscopes by experienced adult gastroenterologists under appropriate pediatric supervision. Procedural success rates, including deep common bile duct cannulation and stone extraction, were comparable between pediatric and adult patients, with no statistically significant differences observed. Importantly, complication rates in children were not higher than those in adults and were generally low, underscoring the safety of ERCP in this younger population when performed in specialized centers. Lower pancreatic duct cannulation, stenting, and biliary stent rates in children likely reflect less complex disease compared to adults. Despite equipment limitations, ERCP is effective for pediatric biliary stones. However, larger multicenter studies and enhanced pediatric training are needed to optimize outcomes. With proper expertise, ERCP is a safe and valuable treatment for pediatric choledocholithiasis.

## Acknowledgments

The authors thank the medical staff of the Adult Gastroenterology Clinic of Ondokuz Mayis University for their support.

## Author contributions

**Conceptualization:** Berk Baş, Ömer Küçükdemirci.

**Data curation:** Berk Baş, Ömer Küçükdemirci.

**Formal analysis:** Berk Baş, Ömer Küçükdemirci.

**Investigation:** Berk Baş.

**Methodology:** Berk Baş.

**Project administration:** Berk Baş.

**Resources:** Berk Baş.

**Supervision:** Berk Baş, Ömer Küçükdemirci, Müge Ustaoğlu, Ahmet Bektaş.

**Validation:** Berk Baş, Ömer Küçükdemirci.

**Writing – original draft:** Ömer Küçükdemirci.

**Visualization:** Müge Ustaoğlu, Ahmet Bektaş.

**Writing – review & editing:** Müge Ustaoğlu, Ahmet Bektaş.
